# Synergistic Effects of Plant Growth Promoting Rhizobacteria and Chitosan on* In Vitro* Seeds Germination, Greenhouse Growth, and Nutrient Uptake of Maize (*Zea mays *L.)

**DOI:** 10.1155/2016/7830182

**Published:** 2016-01-20

**Authors:** Nadège A. Agbodjato, Pacôme A. Noumavo, Adolphe Adjanohoun, Léonce Agbessi, Lamine Baba-Moussa

**Affiliations:** ^1^Laboratoire de Biologie et de Typage Moléculaire en Microbiologie, Faculté des Sciences et Techniques, Université d'Abomey-Calavi, 05 BP 1604 Cotonou, Benin; ^2^Centre de Recherches Agricoles Sud, Institut National des Recherches Agricoles du Bénin, BP 03 Attogon, Benin

## Abstract

This study aimed to assess the effects of three plant growth promoting rhizobacteria (PGPR) and chitosan either singly or in combination on maize seeds germination and growth and nutrient uptake. Maize seeds were treated with chitosan and bacterial solution. The germination and growth tests were carried out in square Petri dishes and plastic pots. The combination chitosan-*A. lipoferum*-*P. fluorescens* has increased the seeds vigor index up to 36.44% compared to the control. In comparison to the control,* P. putida* has significantly improved root weight (44.84%) and germinated seed weight (31.39%) whereas chitosan-*P. putida* has increased the shoot weight (65.67%). For the growth test, the maximal heights (17.66%) were obtained by plants treated with the combination* A. lipoferum-P. fluorescens-P. putida*. Chitosan-*P. fluorescens* induced the highest increases of leaves per plant (50.09%), aerial (84.66%), and underground biomass (108.77%) production. The plants inoculated with* A. lipoferum* had the large leaf areas with an increase of 54.08%, while combinations* P. fluorescens-P. putida* and chitosan*-A. lipoferum* improved the aerial and underground dry matter of plants to 26.35% and 18.18%. The nitrogen content of the plants was increased by chitosan*-A. lipoferum-P. fluorescens-P. putida* with an increasing of 41.61%. The combination of chitosan and PGPR can be used as biological fertilizers to increase maize production.

## 1. Introduction

Nowadays, the use of synthesis products such as mineral fertilizers, pesticides, and growth regulators in agriculture causes a real public health problem. Indeed, the heavy metals contained in these agrochemical products contaminate the groundwater and harvested products. Transferred to humans by feeding and/or direct contact, these heavy metals are reported to be involved in the cancer apparition [1].

So, it is urgent to find alternative agricultural practices that do not use the agrochemical products or significantly limit their use. In this perspective, the use of bioresources such as plant growth promoting rhizobacteria (PGPR) and chitosan (chitin derived) caught the researchers attention, because they have considerable agronomic advantages.

PGPR are soil bacteria capable of colonizing the root systems of many cultures and impact positively the plant physiological process [2]. These bacteria are involved in the plant resistance to biotic and/or abiotic stresses [3]. Though their mechanisms of action are not fully elucidated, PGPR are currently classified into four groups: (i) “biofertilizers” for their ability to solubilize mineral phosphates and to fix atmospheric nitrogen [4], (ii) “phytostimulators” for their ability to produce plant hormones [5], (iii) “rhizoremediators” for their capacity to degrade organic pollutants [6], and at last (iv) “biopesticides” for their ability to produce siderophores, to synthesize antibiotics, enzymes, and/or fungicidal compounds [7]. Noumavo et al. [8] observed a clear improvement of* in vitro* maize seeds germination and greenhouse growth after seeds inoculation by PGPR and their various combinations. These rhizobacteria have strongly produced Indole Acetic Acid (IAA), ammonia (NH_3_), hydrogen cyanide (HCN), and exopolysaccharides and exhibit strong antifungal activity against* Fusarium verticillioides*, real pathogen of maize [9].

Apart from the PGPR, many other biopesticides such as chitosan are able to stimulate plants innate ability to defend against fungal infections [10]. Indeed, chitosan is a natural polymer derived from chitin, commonly founded in the carapace of crustaceans, insect cuticle, and fungi walls [11]. This biopesticide is also a stimulator of flowering and fruition [12] and also used as a plant growth regulator [9].

In this context, the present study aims to assess the effect of three PGPR (*A. lipoferum, P. fluorescens, *and* P. putida*) and chitosan either singly or in combination on* in vitro* seeds germination and greenhouse growth of maize.

## 2. Materials and Methods

### 2.1. Materials

The rhizobacteria strains used (*A. lipoferum, P. fluorescens, *and* P. putida*) were isolated from maize rhizosphere in southern Benin (West Africa) and characterized by Adjanohoun et al. [13]. These bacteria are part of the strains collection of Laboratory of Biology and Molecular Typing in Microbiology (University of Abomey-Calavi, Benin). The chitosan (chitin extracted from exoskeleton of crayfish) used was supplied by the Department of Vegetables Physiology and Biochemistry of the Cuban National Institute of Agricultural Sciences (Cuba, Latin America). The maize (*Zea mays L*.) variety used was EVDT 97 STR C1. It is a composite of a cycle of 85 to 90 days [14]. The substrate used for the greenhouse growth was a deep reddish ferrous soil whose chemical characteristics are presented in Table 1. This substrate is taken at Niaouli village in the district of Allada in Atlantic Department (Benin, West Africa). It is situated at an altitude of 105°, longitude of 2° 19′ east, and latitude of 6° 12′ north. Niaouli is characterized by a maritime subequatorial climate with two rainy seasons (a great season from March to June and a small season from September to November) and two dry seasons (July to September and from November to March). The average pluviometry is 1.200 mm with maximum precipitations in June and October and minimum precipitations in August. The average temperature is around 27°C.

### 2.2. Preparation of PGPR Inoculum and Chitosan Solution

After rhizobacteria revivification in agar medium, the PGPR inoculum was prepared by culture in Mueller-Hinton broth (beef infusion solids, 2.0 g/L; casein hydrolysate, 17.5 g/L; starch, 1.5 g/L; pH 7.4 ± 0.2 at 25°C, SIGMA) for 24 hours at 30°C for* P. fluorescens *and* P. putida* and 37°C for* A. lipoferum*. The bacterial suspensions were centrifuged at 10.000 rpm for 10 min. Pellets obtained were suspended in nutrient broth and the inoculums obtained were adjusted to 1 × 10^8^ CFU/mL (OD 0.45 at 610 nm for* Pseudomonas* and OD 0.5 at 600 nm for* A. lipoferum*) with the spectrophotometer (BioMATE 3S, Thermo scientific) as described by Govindappa et al. [15]. Noting that, for coinoculation of two or three rhizobacteria, each previous inoculum was mixed in the ratio 1 : 1 (v/v) prior to inoculation, the chitosan solution was used at a concentration of 0.5 g/L.

### 2.3. Experimental Design

The experimental design used was a complete randomized block of 16 treatments with 4 repetitions for each treatment. The treatments were defined as follows: CTL: control (*without* bacteria and chitosan); lip: treated only with* A. lipoferum*; flu: treated only with* P. fluorescens*; put: treated only with* P. putida*; lip-flu: treated with* A. lipoferum-P. fluorescens *in the same proportion;* lip*-*put*: treated with* A. lipoferum-P. putida *in the same proportion; flu-put: treated with* P. fluorescens-P. putida *in the same proportion; lip-flu-put: treated with* A. lipoferum-P. fluorescens-P. putida *in the same proportion; Q: treated only with chitosan; Q.lip: treated with chitosan*-A. lipoferum *in the same proportion; Q.flu: treated with chitosan*-P. fluorescens *in the same proportion; Q.put: treated with chitosan*-P. putida *in the same proportion; Q.lip-flu: treated with chitosan*-A. lipoferum-P. fluorescens *in the same proportion; Q.lip-put: treated with chitosan*-A. lipoferum-P. putida *in the same proportion; Q.flu-put: treated with chitosan*-P. fluorescens-P. putida *in the same proportion; Q.lip-flu-put: treated with chitosan*-A. lipoferum-P. fluorescens-P. putida *in the same proportion.

### 2.4. Evaluation of PGPR and Chitosan Effect on* In Vitro* Maize Seeds Germination

#### 2.4.1. Seeds Inoculation with PGPR and Chitosan

The maize seeds were soaked for 2 min in a Sodium Hypochlorite solution (0.024%) and rinsed five times with sterile distilled water [16]. The treated seeds were transferred to the chitosan solution (0.5 g/L) for twelve hours or in a PGPR inoculum (1 × 10^8^ CFU/mL) for 30 min [17] according to the treatments. Regarding PGPR and chitosan combinations, seeds were firstly treated with chitosan before PGPR. Indeed, after soaking in chitosan solution, seeds were transferred into the bacterial solution for 30 minutes.

#### 2.4.2. *In Vitro* Seeds Germination

According to the treatments, 20 inoculated seeds were arranged in an equidistant manner in a square sterile Petri dish (11.8 cm of side) previously wallpapered with towel paper moistened with 10 mL of sterile distilled water. The seeds were again covered with towel paper moistened with 10 mL of sterile distilled water. The Petri dish was closed and incubated at 30°C for 7 days [18].

After germination, the number of germinated seeds per Petri dish was counted in order to determine the germination percentage corresponding to number of germinated seeds/number of seeds set on germination [19]. The root and shoot lengths of each germinated seed were measured to determine the vigor index [(root length + shoot length) × germination percentage] [20]. Using a digital scale (Highland HCB 302, max: 300 g × 0.01 g) the germinated seed (seed-shoot-root), root only, and shoot only were weighed.

### 2.5. Evaluation of the PGPR and Chitosan Effect on Maize Plant Growth

#### 2.5.1. Sowing and Inoculation of Maize Seeds

The substrate (used soil) was sifted with sieve (2 mm of diameter) before being sterilized twice at 120°C for 20 minutes with 24-hour time interval [14]. Twelve (12) kilogrammes of the substrate were then weighed in each pot (13 dm^3^). Each pot was moistened to 2/9th of Maximum Retention Capacity (MRC = 4266.67 mL) of the substrate 24 hours before sowing [21]. MRC is the maximum quantity of water that the substrate can absorb without being flooded.

One seed hole of about 5 cm depth was realized in the centre of each pot. Two (2) maize seeds previously treated with chitosan or no were introduced into the seed hole. These seeds were immediately inoculated with 10 mL of bacterial suspension (1 × 10^8^ CFU/mL) according to each treatment accepted by the control. The seed hole has been closed and the pots were kept in the greenhouse.

#### 2.5.2. Plants Maintenance and Data Collection

The pots were watered to 1/18th of the RMC of substrate daily at 48 hours. Seven Days After Sowing (DAS), the least vigorous of the two plants was removed. The average day and night temperature under greenhouse during the experimentation were 27.89°C and 24.86°C. The data on different growth parameters were collected from 7th to 30th DAS. The height, circumference, and number of leaves per plant were measured every 96-hour time interval. The leaf area was estimated by using Ruget [22] method (*k*  × length × width, where *k* = 0.75). The aerial biomass and underground biomass were collected and weighed (Highland HCB 302, max: 300 g × 0.01 g) at 30th DAS. These types of biomass were dried at 65°C for 72 hours [17] for the determination of the dry matter (% fresh biomass).

### 2.6. Statistical Analysis

The different parameters evaluated were submitted to Analysis Of Variance (ANOVA) at probability level of 0.05 and the Student Newman-Keuls (SNK) tests using the Statistical Analysis System (SAS, Version 8.1) software. In this analysis model, the treatments were considered as a stationary factor while the repetitions were considered as a random factor [23].

In order to subdivide all treatments at the groups containing fairly homogenous elements, a numerical classification based on the evaluated parameters was performed (SAS software) according to the coefficient of determination *R* ^2^ = 0.50 [24]. The groups resulting from numerical classification were put in relation to the different variables through Principal Components Analysis (PCA) with Minitab software (version 14) as described by Uguru et al. [25], to facilitate the interpretation of numerical classification results.

## 3. Results and Discussion

### 3.1. Effects of PGPR and Chitosan on* In Vitro* Maize Germination

Table 2 shows the effects of PGPR, chitosan, and different PGPR-chitosan combinations on the germinative parameters of maize. For each parameter evaluated, the data were varied from one treatment to another. The difference of effect observed was significant (*p* < 0.05) for germination percentage while it was very highly significant (*p* < 0.001) for all other parameters.

**Figure 1:**
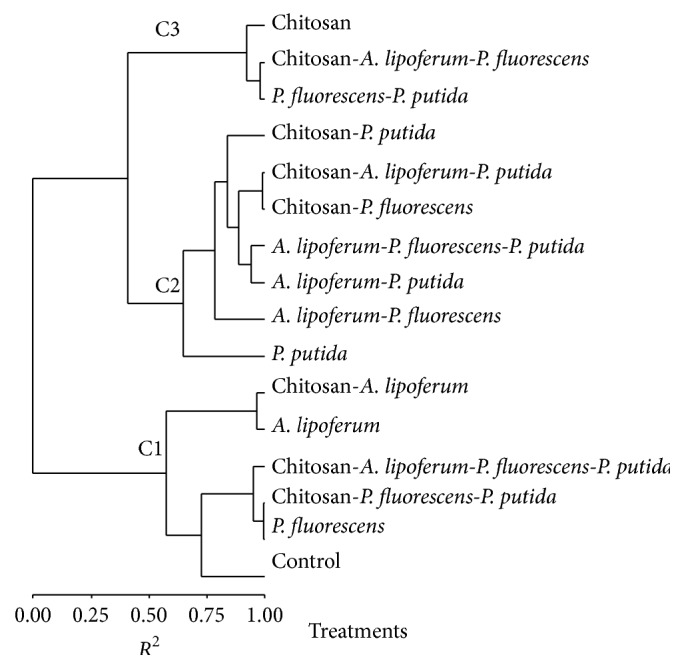
Dendrogram presenting the treatments clusters (C) derived from numerical classification based on treatments effects on the germinative parameters.

The result of the numerical classification of the different treatments (CTL, lip, flu, put, lip-flu, lip-put, flu-put, lip-flu-put, Q, Q.lip, Q.flu, Q.put, Q.lip-flu, Q.lip-put, Q.flu-put, and Q.lip-flu-put) according to their effects on germinative parameters (percentage of germination, shoot and root length, vigor index, shoot and root weight, and weight of germinated seed) is presented as a dendrogram form (Figure 1). Based on the results of numerical classification (dendrogram), the previous treatments had been classified into three clusters. Cluster 1 was composed of flu, lip, Q.lip, Q.flu-put, Q.lip-flu-put, and CTL. Cluster 2 was represented by put, lip-put, lip-flu, lip-flu-put, Q.flu, Q.put, and Q.lip-put. At last, Cluster 3 was composed of flu-put, Q, and Q.lip-flu. The average values of germinative parameters according to each cluster are presented in Table 3.

In order to describe the relationships between different clusters and variables for a clear interpretation of these results, a Principal Component Analysis (PCA) was performed. The PCA results indicated that the first two axes (Axe 1 and Axe 2) were sufficient to explain 100% of the total information. The first principal component (Axe 1) was opposed the germination percentages to the shoot length, shoot weight, root weight, and germinated seed weight. We deduce that the treatments inducting the improvement of maize seed germination have not improved the shoot length, shoot weight, root weight, and germinated seed weight. The characteristics of each treatment cluster were presented as follows.

**Figure 2:**
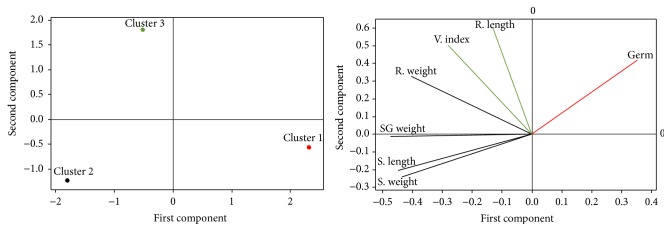
Correlation between treatments (clusters) and germinative parameters according to Principal Components Analysis (PCA): projection of treatments and parameters in factorial axis system. Germ: germination percentage; S: shoot; R: root; V: vigor; SG: seed germinated.

*Characteristics of Cluster 1*. The projection of different variables in the axes system defined by the previous three clusters (Figure 2) revealed that the treatment of Cluster 1 has significantly influenced the maize seeds germination. All seeds were inoculated by* P. putida *and no inoculated seeds (control) were germinated (100%). The results are similar to the 100% obtained by Noumavo et al. [8] with the seeds inoculated by the combination of* P. fluorescens-P. putida*. In our study, the other treatments induced germination percentages ranging from 88.7% to 98.7%. The high germination percentage obtained in our study attests the good viability of the used seeds. Those seeds, after 12 hours of soaking, could absorb enough water to trigger the respiratory and metabolic mechanisms that control the seed germination [26]. It may be also due to the best synthesis of gibberellin, hormone trigger of *α*-amylase, protease, and nuclease activity. These enzymes are necessary to the hydrolysis and assimilation of starch [16].

*Characteristics of Cluster 2*. The treatments of Cluster 2 have induced the better shoot growth and characterized by heavy root, shoot, and seeds germinated. Thus, the highest shoot length was observed on the seeds inoculated with the combinations of* A. lipoferum-P. fluorescens* and chitosan*-P. putida* (9,828 cm). These two treatments have, respectively, increased shoot length to 68.88% and 65.12% compared to control. These increases are superior to the 54.51% obtained by Noumavo et al. [8] with the combination of* A. lipoferum-P. fluorescens* on the same maize variety. Note that only chitosan was induced an increase of shoot length to 23.59%. But this increase is even better when chitosan was combined with a PGPR (65.12% with chitosan-*P. putida*). The treatments* P. putida* and chitosan*-A. lipoferum-P. fluorescens* have, respectively, induced an increase of root weight to 44.84% and 38.10%, while the treatments of chitosan-*P. putida* and* P. putida* improved the shoot weight and germinative percentage, respectively, to 65.67% and 26.60%.

*Characteristics of Cluster 3*. The seeds inoculated with treatment of Cluster 3 are characterized by longest roots, vigorous seeds, and highest percentage of germination. Indeed, the combination of chitosan*-A. lipoferum-P. fluorescens* has induced the longest roots and highest seeds vigor index with an increase, respectively, up to 42.39% and 36.44% compared to control. The improvement of seed vigor index observed in our study by PGPR and chitosan may be due to an induction of better synthesis of cytokines, hormones stimulating cell division [27], and/or auxins, hormones stimulating cell elongation [28].

### 3.2. Effects of PGPR and Chitosan on Maize Growth Parameters

In this study, the effect of rhizobacteria, chitosan, and their combinations on the greenhouse growth of maize plants at 30 JAS was variable (Table 4). The difference of effect between the treatments was significant (*p* < 0.05) for the number of leaves per plant and leaf area.

The combinations of three rhizobacteria (*A. lipoferum.-P. fluorescens-P. putida*) and their combination with chitosan (chitosan-*A. lipoferum-P. fluorescens-P. putida*) have induced the best height growth of maize plants with an increase for about 17% (Table 4). This improvement is similar to the 18.67% obtained by Satrani et al. [29] following the inoculation of* Cedrus atlantica *Manetti seeds with* P. fluorescens* A6RI. The plants inoculated with* A. lipoferum, P. putida,* and the combinations chitosan-*P. fluorescens, *chitosan-*P. fluorescens-P. putida*, and chitosan-*A*.* lipoferum-P. fluorescens-P. putida* had best circumferences.

The treatments* P. fluorescens* and chitosan have improved the number of leaves per maize plant, respectively, to 40.71% and 24.95% compared to the control. The combination of chitosan*-P. fluorescens* has increased the number of leaves for 50.09%. We conclude that in addition to its own potential, the chitosan has boosted the performance of* P. fluorescens*. Ohta et al. [30] showed that the chitosan has the capacity to increase the number and biomass of* Eustoma grandiflorum *flowers. Thus, the results of those authors Ohta et al. [30] are confirmed in this study as we record an increase of maize leaf area (35.34% compared to the control) probably inducted by chitosan. In addition, Hadwiger et al. [31] have shown the effectiveness of chitosan in stimulating the tomato growth. But we should notice that maize plants inoculated with* A. lipoferum *and combination of chitosan*-A. lipoferum-P. fluorescens-P. putida *display the large leaves. Those combinations increased the leaf area, respectively, to 35.34% and 52.41% compared to the control. Nevertheless, these results are lower than the 91.41% obtained by Gholami et al. [16] after inoculating maize seeds with the* A. brasilense* DSM 1690 strain. The plant growth promoting effect inducted by rhizobacteria is then dependent to species and strains of microorganism.

### 3.3. Effects of PGPR and Chitosan on the Biomass Production

A very highly significant difference was observed between the effects induced by rhizobacteria, chitosan, and their combinations for aerial fresh biomass produced by maize plants at 30 DAS in greenhouse conditions (*p* < 0.001). For underground fresh biomass, the difference of effects was highly significant (*p* < 0.01). Contrary to two previous parameters, the rhizobacteria, chitosan, and their combinations had no significant effect on the production of dry matter by the maize plants (*p* > 0.05). The combinations of chitosan-*P. fluorescens* and chitosan-*A. lipoferum* have induced a good production of fresh aerial biomass with respective increases of 84.66% and 64.72% compared to the control. The plants treated with the combination of chitosan-*P. putida* have produced the highest underground fresh biomass, inducing an increase of 108.77% compared to the control (Table 5). The improvements of aerial fresh biomass obtained in this study are higher than those obtained by Noumavo et al. [8] with the same bacteria strains (27.71% for* P. fluorescens* and 39.54% for* A. lipoferum*). We can therefore deduce that there is a synergic effect between chitosan,* P. fluorescens,* and* A. lipoferum* to improve of the maize plants biomass production. The same remark has been done for root growth.

The promoting effects of rhizobacteria and chitosan could result primarily from the induction of a better roots growth, increasing the exchange surface between soil and maize plants. The consequence of large exchange surface is a better nutrition of the plants and a good plant development [29]. Moreover Lemanceau et al. [32] stated that* Pseudomonas* bacteria are involved in the improvement of the plants growth and health. Furthermore, testing the effectiveness of the combination of chitosan and lysozyme on cucumber and tomatoes root rot in greenhouse conditions, Neova Technologies Inc. [33] showed that the combination of chitosan-lysozyme reduces the level lesion of tomato stem to 14% compared to the control. Thus, according to Leclerc et al. [34], this activity of chitosan is due to the synthesis of phytoalexins, chitinases, glucanases, pectinases, and the lignin formation during the plant growth.

### 3.4. Effects of PGPR and Chitosan on Nitrogen, Phosphorus, and Potassium Uptake by the Maize Plants

The effects induced by rhizobacteria and chitosan on nitrogen, phosphorus, and potassium contents in maize plants after 30 DAS are presented in Table 6. Apart from the treatment* P. fluorescens*, all other treatments have significantly improved the nitrogen content in the aerial biomass of maize plants. Considering the root recorded parameter, only the root of the plants treated with* A. lipoferum* and their combination with chitosan displays the highest levels of nitrogen compared to the control. Thus, the nitrogen content in the aerial part of the maize plants was increased up to 41.61% compared to the control, after inoculation with the combination of the three rhizobacteria and chitosan (chitosan-*A. lipoferum-P. fluorescens-P. putida*). The nitrogen content in the roots of maize plants was increased up to 19.51% after inoculation with* A. lipoferum* and the combination of chitosan*-A. lipoferum*. The treatments of* P. fluorescens *and* P. putida* and the combinations of* A. lipoferum-P. fluorescens* and chitosan-*A. lipoferum-P. fluorescens-P. putida* have induced an increase of potassium content in the aerial part of maize plants. The potassium content in root plants was increased by the combinations of chitosan-*A. lipoferum-P. fluorescens* and chitosan-*A. lipoferum-P. putida*. Thus, the potassium content in the aerial and underground part of maize plant was, respectively, increased up to 6.34% and 27.16%. Overall, we hold back that several treatments have induced an improvement of the mineral nutrition of maize plants specially nitrogen and potassium nutrition.

These previous results are very interesting, because the nitrogen, potassium, and phosphorus are the first three major elements that plant needs in highest quantities for good nutrition. That is why they are included in the composition of majority chemical fertilizers. Indeed, nitrogen is the main limiting nutrient for the plant growth [35]. It is an essential constituent of nucleotides, membrane lipids, and amino acids (enzymatic and structural proteins). The phosphorus plays a major role in photosynthesis, respiration, storage and energy transfer, division, and elongation [36]. It is requisite for seed formation, which contains the highest phosphorus content of the plant. This nutritive element is essential for the flowering, fruit setting, fruit swelling, and seeds maturation. Regarding the potassium, it involves root development, absorption of cations (NH^4+^, Ca^2+^, Mg^2+^, Cu^2+^, and Fe^2+^), accumulation of protein hydrates, activation of photosynthetic enzymes, conservation of cell turgescence, and stomates regulation. Potassium is also an element involved in plants resistance to frost, drought, and disease. It is also essential for the transfer of assimilates to storage organs (bulbs and tubers). In a study conducted by Biari et al. [37], the improvement of seeds yields of maize plants inoculated with microorganisms was associated with an increase of nitrogen, phosphorus, potassium, iron, zinc, manganese, and copper absorption.

## 4. Conclusion

This study confirmed the potential of the three studied rhizobacteria (*A. lipoferum, P. fluorescens,* and* P. putida*) and the chitosan to promote seed germination and vegetative growth of maize plants. Indeed, the germinative and growth parameters of maize have been improved both individually and in combination by the chitosan and rhizobacteria. For most evaluated parameters, the combination of chitosan and the rhizobacteria was better than the chitosan only. Thus, the combination of chitosan and plant growth promoting rhizobacteria can be used as biofertilizers to improve the maize production.

## Figures and Tables

**Table 1:** Chemical properties of used soil for maize growth.

Sample	pH		Assimilable phosphorus (ppm)	Organic carbon (%)	Organic matter (%)	Exchangeable bases (meq/100 g)			
	Water	kcl				K	Ca	Mg	Na
Soil	5.6	4.9	8	0.70	1.21	0.14	2.87	0.81	0.18

ppm: parts per million; meq: milliequivalents.

**Table 2:** Effect of *A. lipoferum*, *P. fluorescens*, *P. putida,* and chitosan on the maize germinative parameters.

Treatments	Germination (%)		Shoot length (cm)		Root length (cm)		Vigor index		Shoot weight (mg)		Root weight (mg)		Germinated seed weight (mg)	
	m	*σ*	m	*σ*	m	*σ*	m	*σ*	m	*σ*	m	*σ*	m	*σ*
CTL	100^a^	0.0	5.952^e^	0.452	12.736^c^	1.127	1868.8^b^	110.8	0.233^c^	0.026	0.252^cd^	0.009	0.876^ef^	0.055
lip	98.7^ab^	2.3	8.072^abcd^	0.801	13.822^abc^	0.861	2162.3^ab^	62.1	0.307^abc^	0.043	0.226^d^	0.029	0.915^cdef^	0.025
flu	95.0^ab^	0.0	6.879^ced^	0.589	16.146^abc^	0.541	2187.4^ab^	35.5	0.235^c^	0.018	0.261^cd^	0.024	0.889^def^	0.023
put	100^a^	0.0	8.961^abc^	1.33	13.306^bc^	0.49	22.267^ab^	231	0.375^a^	0.038	0.365^a^	0.061	1.151^a^	0.07
lip-flu	88.7^b^	7.9	10.052^a^	1.553	16.806^abc^	1.998	2363^a^	141.1	0.34^ab^	0.057	0.315^abc^	0.024	1.032^abcde^	0.032
lip-put	96.2^ab^	2.3	8.659^abcd^	0.848	15.346^abc^	2.116	2313.1^a^	206.2	0.333^abc^	0.051	0.342^ab^	0.013	1.109^ab^	0.048
flu-put	95.0^ab^	3.7	8.612^abcd^	0.773	18.001^a^	3.361	2529.5^ab^	301.5	0.303^abc^	0.024	0.336^ab^	0.059	1.048^abcd^	0.068
lip-flu-put	96.2^ab^	4.4	9.694^a^	1.288	16.425^abc^	0.938	2509.8^a^	133.2	0.34^ab^	0.047	0.312^abc^	0.007	1.055^abcd^	0.064
Q	98.7^ab^	2.3	7.356^bcde^	0.353	17.669^ab^	0.642	2470.7^a^	84.6	0.265^abc^	0.018	0.313^abc^	0.018	0.978^bcde^	0.035
Q.lip	98.7^ab^	2.3	8.09^abcd^	0.765	12.798^c^	0.383	2062.2^ab^	41.7	0.314^abc^	0.038	0.256^cd^	0.016	1.039^abcde^	0.052
Q.flu	96.2^ab^	4.4	9.219^ab^	0.706	14.367^abc^	1.541	2267.4^ab^	144.8	0.316^abc^	0.039	0.315^ab^	0.071	0.999^abcd^	0.123
Q.put	92.5^ab^	5.5	9.828^a^	0.868	13.071^c^	3.017	2125.9^ab^	315	0.386^a^	0.05	0.261^cd^	0.007	1.076^abc^	0.06
Q.lip-flu	97.5^ab^	2.6	8.025^abcd^	0.969	18.135^a^	1.206	2549.9^a^	215.5	0.298^abc^	0.022	0.348^ab^	0.013	0.99^abcde^	0.051
Q.lip-put	95.0^ab^	3.7	8.822^abcd^	1.932	14.021^abc^	1.659	2165.6^ab^	265.4	0.32^c^	0.088	0.282^bcd^	0.041	0.967^bcde^	0.174
Q.flu-put	97.5^ab^	2.6	6.643^de^	0.383	15.442^abc^	1.218	2155.5^ab^	198	0.237^abc^	0.014	0.263^cd^	0.018	0.896^def^	0.028
Q.lip-flu-put	97.5^a^	4.6	7.227^bcde^	0.414	16.122^abc^	1.592	2273.5^ab^	182.9	0.24^c^	0.009	0.225^d^	0.009	0.809^f^	0.003

Signification	*∗*		*∗∗∗*		*∗∗∗*		*∗∗∗*		*∗∗∗*		*∗∗∗*		*∗∗∗*	

^*∗*^ *p* < 0.05 (significant); ^*∗∗∗*^ *p* < 0.001 (very highly significant); m: mean; *σ*: standard error. In a column, the means with different letters are significantly different than the probability level of 5% according to Student Newman-Keuls test. CTL: control (without bacteria and chitosan); lip: treated only with *A. lipoferum; *flu: treated only with *P. fluorescens; *put: treated only with* P. putida; *lip-flu: treated with *A. lipoferum-P. fluorescens *in the same proportion; *lip*-*put*: treated with *A. lipoferum-P. putida *in the same proportion; flu-put: treated with *P. fluorescens-P. putida *in the same proportion; lip-flu-put: treated with *A. lipoferum-P. fluorescens-P. putida *in the same proportion*;* Q: treated only with chitosan; Q.lip: treated with chitosan*-A. lipoferum *in the same proportion*; *Q.flu: treated with chitosan*-P. fluorescens *in the same proportion; Q.put: treated with chitosan*-P. putida *in the same proportion*; *Q.lip-flu: treated with chitosan*-A. lipoferum-P. fluorescens *in the same proportion; Q.lip-put: treated with chitosan*-A. lipoferum-P. putida *in the same proportion; Q.flu-put: treated with chitosan*-P. fluorescens-P. putida *in the same proportion*; *Q.lip-flu-put: treated with chitosan*-A. lipoferum-P. fluorescens-P. putida *in the same proportion.

**Table 3:** Average means of germinative parameters according to cluster groups.

Clusters	Treatments	Parameters						
		Germination (%)	Shoot length (cm)	Root length (cm)	Vigor index	Shoot weight (mg)	Root weight (mg)	Germinated seed weight (mg)
Cluster 1	flu, Q.flu-put, lip, Q.lip, Q.lip-flu-put and CTL.	0.98	7.14	14.52	21.18	0.26	0.25	0.91
Cluster 2	Q.flu, Q.lip-put, lip-put, lip-flu-put, Q.put, lip-flu and put.	0.95	9.32	14.77	22.82	0.35	0.31	1.06
Cluster 3	flu-put, Q.lip-flu and Q	0.98	8.00	17.94	25.17	0.29	0.33	1.01

CTL: control (without bacteria and chitosan); lip: treated only with *A. lipoferum; *flu: treated only with *P. fluorescens; *put: treated only with* P. putida; *lip-flu: treated with *A. lipoferum-P. fluorescens *in the same proportion; *lip*-*put*: treated with *A. lipoferum-P. putida *in the same proportion; flu-put: treated with *P. fluorescens-P. putida *in the same proportion; lip-flu-put: treated with *A. lipoferum-P. fluorescens-P. putida *in the same proportion*;* Q: treated only with chitosan; Q.lip: treated with chitosan*-A. lipoferum *in the same proportion*; *Q.flu: treated with chitosan*-P. fluorescens *in the same proportion; Q.put: treated with chitosan*-P. putida *in the same proportion*; *Q.lip-flu: treated with chitosan*-A. lipoferum-P. fluorescens *in the same proportion; Q.lip-put: treated with chitosan*-A. lipoferum-P. putida *in the same proportion; Q.flu-put: treated with chitosan*-P. fluorescens-P. putida *in the same proportion*; *Q.lip-flu-put: treated withchitosan*-A. lipoferum-P. fluorescens-P. putida *in the same proportion.

**Table 4:** Effects of *A. lipoferum*, *P. fluorescens*, *P. putida,* and chitosan on growth parameters of maize plants at 30 DAS.

Treatments	Height (cm)		Leaves/plant		Circumference (cm)		Leaf area (cm^2^)	
	m	*σ*	m	*σ*	m	*σ*	m	*σ*
CTL	23.50^a^	1.94	5.33^b^	0.51	3.16^a^	0.51	100.13^b^	12.50
lip	26.95^a^	4.00	7.00^ab^	0.75	4.00^a^	0.75	154.28^a^	16.93
flu	24.35^a^	0.50	7.50^ab^	1.19	3.80^a^	1.19	126.83^ab^	8.26
put	23.95^a^	1.65	6.50^ab^	0.53	3.90^a^	0.53	123.48^ab^	4.40
lip-flu	24.90^a^	0.42	6.75^ab^	1.16	3.67^a^	1.16	141.24^ab^	13.39
lip-put	26.05^a^	2.22	6.75^ab^	0.88	3.67^a^	0.88	140.89^ab^	12.15
flu-put	23.50^a^	2.53	6.25^ab^	0.88	3.32^a^	0.88	134.90^ab^	4.34
lip-flu-put	27.65^a^	2.77	7.25^ab^	1.38	3.55^a^	1.38	141.21^ab^	37.41
Q	25.63^a^	1.08	6.66^ab^	0.51	3.53^a^	0.51	135.53^ab^	10.94
Q.lip	25.92^a^	3.25	6.50^ab^	1.19	3.65^a^	1.19	147.55^a^	13.62
Q.flu	26.32^a^	2.10	8.00^a^	1.30	3.90^a^	1.30	127.11^ab^	8.84
Q.put	24.13^a^	0.44	6.66^ab^	0.51	3.63^a^	0.51	135.84^ab^	6.73
Q.lip-flu	25.07^a^	0.81	6.50^ab^	0.53	3.82^a^	0.53	136.84^ab^	7.89
Q.lip-put	23.67^a^	0.49	6.75^ab^	0.88	3.75^a^	0.88	135.17^ab^	3.63
Q.flu-put	24.17^a^	0.78	6.75^ab^	0.46	3.90^a^	0.46	136.84^ab^	3.71
Q.lip-flu-put	27.42^a^	2.96	7.50^ab^	0.53	3.92^a^	0.53	152.71^a^	27.22

Signification	ns		*∗*		ns		*∗*	

^ns^ *p* > 0.05 (not significant); ^*∗*^ *p* < 0.05 (significant); m: mean; *σ*: standard error. In a column, the means with different letters are significantly different at the probability level of 5% according to Student Newman-Keuls test. CTL: control (without bacteria and chitosan); lip: treated only with *A. lipoferum; *flu: treated only with *P. fluorescens; *put: treated only with* P. putida; *lip-flu: treated with *A. lipoferum-P. fluorescens *in the same proportion; *lip*-*put*: treated with *A. lipoferum-P. putida *in the same proportion; flu-put: treated with *P. fluorescens-P. putida *in the same proportion; lip-flu-put: treated with *A. lipoferum-P. fluorescens-P. putida *in the same proportion*;* Q: treated only with chitosan; Q.lip: treated with chitosan*-A. lipoferum *in the same proportion*; *Q.flu: treated with chitosan*-P. fluorescens *in the same proportion; Q.put: treated with chitosan*-P. putida *in the same proportion*; *Q.lip-flu: treated with chitosan*-A. lipoferum-P. fluorescens *in the same proportion; Q.lip-put: treated with chitosan*-A. lipoferum-P. putida *in the same proportion; Q.flu-put: treated with chitosan*-P. fluorescens-P. putida *in the same proportion*; *Q.lip-flu-put: treated withchitosan*-A. lipoferum-P. fluorescens-P. putida *in the same proportion.

**Table 5:** Effects of *A. lipoferum*, *P. fluorescens*, *P. putida,* and chitosan on biomass and dry matter produced by the maize plant at 30 DAS.

Treatments	Aerial biomass (g)		Underground biomass (g)		Aerial dry matter (%)		Underground dry matter (%)	
	m	*σ*	m	*σ*	m	*σ*	m	*σ*
CTL	15.39^c^	0.26	7.68^c^	1.16	8.99^a^	0.86	7.60^a^	1.21
lip	24.46^ab^	0.47	10.45^abc^	2.03	10.24^a^	1.37	7.06^a^	1.94
flu	20.97^bc^	0.16	10.21^abc^	2.15	8.30^a^	0.62	5.91^a^	1.22
put	17.46^bc^	0.19	10.85^abc^	0.94	9.68^a^	1.01	6.34^a^	0.92
lip-flu	18.68^bc^	0.07	8.71^bc^	1.56	9.75^a^	0.42	7.68^a^	1.51
lip-put	19.92^bc^	0.11	15.02^ab^	3.26	10.75^a^	0.34	7.55^a^	1.87
flu-put	19.04^bc^	0.08	14.02^abc^	3.71	11.35^a^	0.79	6.77^a^	1.09
lip-flu-put	19.82^bc^	0.35	12.03^abc^	3.31	10.18^a^	0.97	6.68^a^	0.77
Q	18.37^bc^	0.16	10.94^abc^	2.17	10.55^a^	1.29	8.75^a^	2.13
Q.lip	25.35^ab^	0.52	7.92^bc^	1.22	10.82^a^	2.50	8.98^a^	2.79
Q.flu	28.42^a^	0.29	12.45^abc^	2.87	9.90^a^	1.68	8.93^a^	2.53
Q.put	20.18^bc^	0.34	16.04^a^	5.10	11.26^a^	1.04	5.46^a^	0.67
Q.lip-flu	18.93^bc^	0.12	9.58^abc^	1.78	10.41^a^	0.27	6.38^a^	1.34
Q.lip-put	17.16^bc^	0.25	8.92^abc^	0.89	9.96^a^	1.21	7.15^a^	1.40
Q.flu-put	18.60^bc^	0.18	11.40^abc^	1.50	10.63^a^	0.89	7.47^a^	1.09
Q.lip-flu-put	20.86^bc^	0.25	11.24^abc^	2.88	9.04^a^	0.83	7.20^a^	1.32

Signification	*∗∗∗*		*∗∗*		ns		ns	

^ns^ *p* > 0.05 (not significant); ^*∗∗*^ *p* < 0.01 (highly significant); ^*∗∗∗*^ *p* < 0.001 (very highly significant) m: mean; *σ*: standard error. In a column, the means with different letters are significantly different at the probability level of 5% according to Student Newman-Keuls test. CTL: control (without bacteria and chitosan); lip: treated only with *A. lipoferum; *flu: treated only with *P. fluorescens; *put: treated only with* P. putida; *lip-flu: treated with *A. lipoferum-P. fluorescens *in the same proportion; *lip-put*: treated with *A. lipoferum-P. putida *in the same proportion; flu-put: treated with *P. fluorescens-P. putida *in the same proportion; lip-flu-put: treated with *A. lipoferum-P. fluorescens-P. putida *in the same proportion*;* Q: treated only with chitosan; Q.lip: treated with chitosan*-A. lipoferum *in the same proportion*; *Q.flu: treated with chitosan*-P. fluorescens *in the same proportion; Q.put: treated with chitosan*-P. putida *in the same proportion*; *Q.lip-flu: treated with chitosan*-A. lipoferum-P. fluorescens *in the same proportion; Q.lip-put: treated with chitosan*-A. lipoferum-P. putida *in the same proportion; Q.flu-put: treated with chitosan*-P. fluorescens-P. putida *in the same proportion*; *Q.lip-flu-put: treated with chitosan*-A. lipoferum-P. fluorescens-P. putida *in the same proportion.

**Table 6:** Effects of *A. lipoferum*, *P. fluorescens*, *P. putida,* and chitosanon nutrients content of maize plants at 30 DAS.

Content of maize plants (% dry matter)						
Treatments	Nitrogen (N)		Phosphorus (P)		Potassium (K)	
	AB	UB	AB	UB	AB	UB
CTL	3.10	3.69	0.14	0.11	6.62	2.43
lip	3.97	4.41	0.14	0.09	6.38	2.10
flu	3.10	3.96	0.14	0.09	7.04	2.59
put	3.98	3.88	0.15	0.10	6.75	2.42
lip-flu	3.25	2.81	0.15	0.08	6.70	2.35
lip-put	3.48	3.08	0.13	0.07	6.01	2.33
flu-put	2.94	3.50	0.14	0.08	6.25	2.43
lip-flu-put	3.33	3.58	0.15	0.10	6.28	2.04
Q	3.42	3.22	0.14	0.10	5.73	2.21
Q.lip	3.79	4.08	0.12	0.12	6.08	2.31
Q.flu	4.16	2.53	0.14	0.09	6.08	2.00
Q.put	3.79	2.59	0.12	0.09	6.74	2.34
Q.lip-flu	4.02	3.30	0.14	0.12	5.74	3.09
Q.lip-put	4.17	3.88	0.15	0.10	6.32	2.82
Q.flu-put	3.24	2.91	0.08	0.07	3.78	1.13
Q.lip-flu-put	4.39	3.51	0.12	0.08	6.77	1.74

AB: aerial biomass; UB: underground biomass; m: mean; *σ*: standard error; CTL: control (without bacteria and chitosan); lip: treated only with *A. lipoferum; *flu: treated only with *P. fluorescens; *put: treated only with* P. putida; *lip-flu: treated with *A. lipoferum-P. fluorescens *in the same proportion; *lip-put*: treated with *A. lipoferum-P. putida *in the same proportion; flu-put: treated with *P. fluorescens-P. putida *in the same proportion; lip-flu-put: treated with *A. lipoferum-P. fluorescens-P. putida *in the same proportion*;* Q: treated only with chitosan; Q.lip: treated with chitosan*-A. lipoferum *in the same proportion*; *Q.flu: treated with chitosan*-P. fluorescens *in the same proportion; Q.put: treated with chitosan*-P. putida *in the same proportion*; *Q.lip-flu: treated with chitosan*-A. lipoferum-P. fluorescens *in the same proportion; Q.lip-put: treated with chitosan*-A. lipoferum-P. putida *in the same proportion; Q.flu-put: treated with chitosan*-P. fluorescens-P. putida *in the same proportion; Q.lip-flu-put: treated with chitosan*-A. lipoferum-P. fluorescens-P. putida *in the same proportion.
